# Old London Houses

**Published:** 1887-07-09

**Authors:** 


### OLD LONDON HOUSES.

MR. CHARLES HAWKINS, in a letter to the *Daily News* under the above heading, gives some very interesting particulars about the Hunters, and other eminent physicians and surgeons. Mr. Hawkins says :—

"In the *Daily News* of the 7th Mr. Hollingshead describes a house in Great Windmill Street as 'the deceased Café de l'Etoile.' Would you grant me space to give the history of this house, which to the medical world is an interesting one? In 1764 Dr. William Hunter, the elder brother of John Hunter, had commenced teaching anatomy in Covent Garden to a society of navy surgeons in the evening, but Garrick at that time appearing at another theatre was a rival that Hunter could not compete with, and he was obliged to lecture in the afternoon. In this school he taught with great success, and attained a great reputation as a surgeon as well as a physician, and having possessed himself of a considerable fortune, in 1765 he applied to the Government to have granted to him a piece of ground that he might at his own expense erect a building fit for a school of anatomy, and supplied a plan 'for a museum in London for the improvement of anatomy, surgery, and physic.' Upon this offer being declined, he commenced building the 'Hunterian School' in Great Windmill Street, comprising a mansion (the deceased Café de l'Etoile), a museum and anatomical department. He took possession of these buildings in 1768, and he lived in the mansion until his death in 1783. These buildings contained the magnificent collection, said to have cost upwards of £100,000, which he bequeathed to the University of Glasgow. Besides the scientific department there were pictures, prints, manuscripts, coins, etc. Upon his death his nephew, Dr. Matthew Baillie, the celebrated physician, went to reside in the house, which he did until 1799. During this time he was physician to St. George's Hospital, and enjoyed the largest physician's practice on record. The house was afterwards inhabited by Mr. James Wilson, the celebrated anatomist, who subsequently sold the property, reserving the 'school' buildings, which were at the rear of the house, and were demolished a few months ago. In 1812 Sir Charles Bell describes them in a letter to his brother, published in 1870 :—'It would delight you to see me the proprietor of this museum, which looks great even now in its greatest confusion—a noble room nobly filled ; it is a room admired for its proportions of great size, with a handsome gallery running round ; the class-room door opens from the gallery. It would require a month to go round with a book in your hand ;' and adds, 'you see me then, my dear George, at the height of my ambition in regard to teaching.' In this school taught William Hunter, Cruikshank, Hewson, Matthew Baillie, James Wilson, Benjamin Brodie, Charles Bell, Hubert Mayo, and Cæsar Hawkins — a great phalanx of great men. It lasted as a school for about sixty years. Some years ago I was a student in this school, and may be pardoned for attempting to rescue its history from simply being known as a deceased foreign café. As for 'the antiquarian pothouse called the Robin Hood,' it outlived the Hunterian School buildings, it being cleared away only a month ago. It appeared to me to have been built about 200 years ago. Great Windmill Street was once in a fashionable neighbourhood. Sir John Shadwell, the son of the Poet Laureate, resided there in 1735 and died there in 1747. He was physician to Queen Anne, George I, and George II. I have thought that 'the antiquarian pothouse' might have been his residence. It well represented a good house of that period. Dr. Denman, the father of the first Lord Denman, lived in Queen (now Denman) Street, leading out of Great Windmill Street. Dr. Willis, said to have had the largest practice as a physician of his day, who attended the children of James II when Duke of York, lived hard by St. Martin's Lane, where he died in 1675."

